# A larger brain confers a benefit in a spatial mate search learning task in male guppies

**DOI:** 10.1093/beheco/aru227

**Published:** 2014-12-24

**Authors:** Alexander Kotrschal, Alberto Corral-Lopez, Mirjam Amcoff, Niclas Kolm

**Affiliations:** ^a^Department of Animal Ecology, Evolutionary Biology Centre, Uppsala University, Norbyvägen 18D, SE-75236 Uppsala, Sweden and; ^b^Department of Zoology/Ethology, Stockholm University, Svante Arrheniusväg 18 B, SE-10691 Stockholm, Sweden

**Keywords:** brain size, cognition, guppy, maze.

## Abstract

Brain size varies dramatically among vertebrates, and selection for increased cognitive abilities is thought to be the key force underlying the evolution of a large brain. Indeed, numerous comparative studies suggest positive relationships between cognitively demanding aspects of behavior and brain size controlled for body size. However, experimental evidence for the link between relative brain size and cognitive ability is surprisingly scarce and to date stems from a single study on brain size selected guppies (*Poecilia reticulata*), where large-brained females were shown to outperform small-brained females in a numerical learning assay. Because the results were inconclusive for males in that study, we here use a more ecologically relevant test of male cognitive ability to investigate whether or not a relatively larger brain increases cognitive ability also in males. We compared mate search ability of these artificially selected large- and small-brained males in a maze and found that large-brained males were faster at learning to find a female in a maze. Large-brained males decreased the time spent navigating the maze faster than small-brained males and were nearly twice as fast through the maze after 2 weeks of training. Our results support that relatively larger brains are better also for males in some contexts, which further substantiates that variation in vertebrate brain size is generated through the balance between energetic costs and cognitive benefits.

## Lay Summary

Does a large brain make you smarter? If you are a guppy male searching for a female in a maze, it does. The association between brain size and smartness is a debated issue, largely due to the lack of experimental data. We compared guppies artificially bred for large and small brains and found that large-brained males learned the route through a spatial maze faster. These results thus support a link between brain size and smartness.

## INTRODUCTION

Because investment into large brains is highly costly (Aiello and Wheeler 1995; Pitnick et al. 2006; Isler and van Schaik 2009; Kotrschal et al. 2013a), the evolution of larger brains is suspected to be primarily driven by selection for increased cognitive ability (Jerison 1973; Dunbar 1998; Striedter 2005). However, to assess the relationship between brain size and cognitive ability in a standardized experiment across species is difficult because every species has evolved to be adapted to its unique ecology (Shettleworth 2009, but see MacLean et al. 2014).

Within-species comparisons of cognitive ability and brain size can circumvent the problems with divergent ecologies. For instance, during the beginning of the 20th century, studies of postmortem brain weight in relation to inferred intelligence in groups of eminent scientists suggested that greater intelligence was associated with greater brain weight (Spitzka 1903). Moreover, in the late 20th century, several studies found similar results when comparing human intelligence test scores and head circumference (reviewed in Rushton and Davison 1996, but see also Witelson et al. 2006).

Lately, comparative analyses have used an indirect approach to investigate the association between brain size and cognitive ability across species. By relating proxies of cognitive ability to brain size, numerous studies have found positive relationships between brain size and behaviors believed to be connected to cognitive ability. For instance, brain size was found to positively correlate with cognitively demanding behaviors such as tool use in birds (Tebbich and Bshary 2004), brood care in cichlids (Gonzalez-Voyer et al. 2008), and bower complexity in bower birds (Madden 2001). Furthermore, large-brained bird species show higher survival in the wild (Sol et al. 2007) and are better at colonizing urban environments (Maklakov et al. 2011; Husby and Husby 2014), whereas large-brained mammals are more likely to establish viable populations after introduction events (Sol et al. 2008). Recently, a large-scale comparative study on self-control, the ability to inhibit a prepotent but ultimately counterproductive behavior, found a striking association between this complex cognitive trait and relative brain size in both birds and mammals (MacLean et al. 2014). Taken together, the aforementioned comparative studies suggest that a large brain confers greater cognitive ability. However, correlative comparative analyses cannot infer causality as the observed patterns may be driven by some third, unknown factor (Martins 2000). Artificial selection, on the other hand, is a powerful tool to provide experimental evidence for the causal relationship between a relatively large brain and higher cognitive abilities (Roderick et al. 1976; Falconer and Mackay 1996).

Here, we investigated the association between relative brain size and cognitive ability using recently developed artificial selection lines of large- and small-brained guppies. These lines had been selected for either large or small relative brain size over 2 generations leading to a divergence of 9% in relative brain size and significant differences in female numerical learning ability between the lines (Kotrschal et al. 2013a, 2013b). In that numerical learning test, individuals were first conditioned to associate a certain number of symbols (2 or 4) with a food reward. Then the symbols were presented without a food reward and the animals indicated by swimming toward one or the other number of symbols whether they had learnt to discriminate between them. Although large-brained females outperformed small-brained females, an effect of relative brain size on male cognitive ability was not evident (Kotrschal et al. 2013a). This sex-specific response may be due to 2 alternative explanations. First, relative brain size may not reflect cognitive ability in males to the same extent as in females (Gahr 1994). Second, the design of the previous cognitive test may have been more suitable for testing female cognitive ability. Here, we test those alternative hypotheses to clarify whether relative brain size is cognitively advantageous for both sexes.

In the guppy, females are more active and innovative while foraging (Laland and Reader 1999), most likely reflecting the fact that female reproductive success is mainly food limited, whereas males are limited by their access to females (Houde 1997). Although food may be a suitable reward for female guppies, access to females should be a more appropriate reward when measuring guppy male cognitive ability. Because male guppies usually utilize large home ranges during mate search and foraging (Croft et al. 2003), spatial cognition is a highly relevant cognitive trait in male guppies. To test whether relatively larger brains confer a cognitive advantage for male guppies, we therefore assessed their ability to navigate a maze using an associative learning regime with access to a female as reward at the end of the maze. Results of a previous study suggested that male guppies with large telencephalon exhibit slower reaction time but make fewer errors in a spatial memory test at least in some populations (Burns and Rodd 2008). Based on the results of Burns and Rodd (2008) in combination with our earlier study showing that large-brained females learned faster than small-brained females (Kotrschal et al. 2013a), we predict that large-brained males will exhibit slower search times in the beginning of the experiment but will learn at a faster rate than their small-brained counterparts.

## METHODS

### Directional selection on brain weight

We examined the relationship between brain size and cognitive ability in laboratory lines of Trinidadian guppies that were artificially selected for large or small relative brain size (Kotrschal et al. 2012, 2013a). Briefly, these selection lines were generated using a standard bidirectional artificial selection design that consisted of 2 replicated treatments (3 up-selected lines and 3 down-selected lines). Because brain size can only be quantified after dissection, we allowed pairs to breed at least 2 clutches first and then we sacrificed the parents for brain quantification and used the offspring from parents with large or small relative brain size as parents for the next generation. More specifically, to select for relative brain size, we selected on the residuals from the regression of brain size (weight) on body size (length) of both parents. We started with 3 times 75 pairs (75 pairs per replicate) to create the first 3 “up-selected” and “down-selected” lines (6 lines in total). We summed up the male and female residuals for each pair and used offspring from the top and bottom 20% of these to form the next-generation parental groups. This means that we used the offspring (2 males and 2 females) of the 15 pairs with the largest residual sums for up-selection and of the 15 pairs with the smallest residual sums for down-selection for each generation. To avoid inbreeding, full-siblings were never mated. See Kotrschal et al. (2013a) for full details on the selection experiment. The selection lines differed in relative brain size by 9% in F2 (Kotrschal et al. 2013a), and body size did not differ between the lines. The realized heritability of male relative brain size was 0.45 (between 0.33 and 0.59) (Kotrschal et al. 2013a, 2014). For the current experiment, we used F3 fish. Prior to the experiment, all fish were removed from their parental tanks after birth, separated by sex at the first onset of sexual maturation and then kept in single-sex groups with a maximum density of 5 individuals in 3-L tanks containing 2cm of gravel with continuously aerated water. We allowed for visual contact between the tanks. The laboratory was maintained at 26 °C with a 12:12h light:dark schedule. Fish were fed a diet of flake food and freshly hatched brine shrimp 6 days per week. All experiments were done blindly because only running numbers identified tanks. We used 36 males randomly chosen from the 3 up- and 3 down-selected lines (6 per line).

### Maze learning

**Figure 1:**
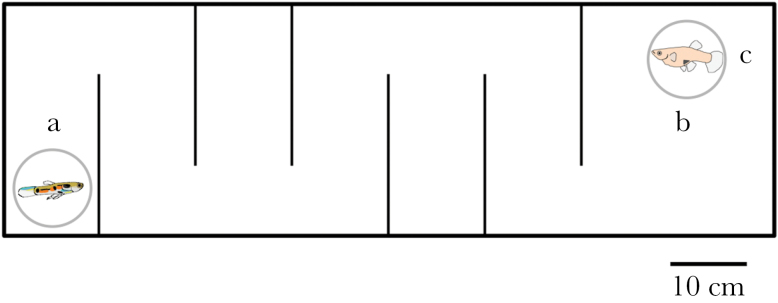
Maze setup used to determine the decrease in search time in male guppies artificially selected for large and small relative brain size. Males were placed in a clear Perspex ring (a); after the ring was remotely lifted, the fish were free to explore the maze. On arrival in the end compartment (b), the clear Perspex ring around the female (c) was remotely lifted. The maze is to scale (scale bar represents 10cm) while fish are depicted approximately 5 times larger.

In order to test whether relatively larger brains confer a cognitive advantage for male guppies, we assessed their ability to decrease the time spent navigating a maze using an associative learning regime with access to a female as reward at the end of the maze. As explained above, spatial cognition should be a highly relevant cognitive trait in male guppies (Croft et al. 2003). Groups of females have smaller home ranges and are repeatedly visited by males (Houde 1997). Our setup mimicked this situation in which males need to navigate through shallow water in the search of females. The maze was constructed of a 100×30cm tank with opaque walls, the bottom was covered by fine gravel and the water depth was 10cm. The maze also contained 2 dead-end areas to increase the level of difficulty and counteract potential biases from differences in overall swimming activity levels (Figure 1). Once per day, each fish was placed into a clear Perspex ring (Figure 1a) at one end of the maze. After a 1-min acclimation period, the ring was lifted and the time until the fish left the start compartment was recorded (“latency” from hereon). Next, the time until the fish had reached the end compartment was recorded (“search time” from hereon, Figure 1b). On arrival of the focal male in the final compartment, another clear Perspex ring was remotely lifted (Figure 1c), which released a “receptive” female as a reward (see below). The direct line distance from start to finish was 100cm. Average critical swimming speed (the maximal speed animals can swim with for extended periods of time) for males of this population of guppies is 13cm/s (Lievens 2012). Individuals swimming on the shortest path should, therefore, be able to complete the maze within 100/13 = 7.7 s. After completion of the maze, the male and female were allowed to interact for 3min. If the focal male did not complete the maze within 8min, it was gently guided through the maze with a hand net. To ensure the potential for positive associative learning also for the males that did not complete the maze by themselves, they were also rewarded with access to the female, but they were treated as missing values during analyses. The order of males during the day was random with regard to brain size. We kept the sequence during the experiment to ensure a constant time period of approximately 24h until the same individual was retested. Female guppies actively solicit copulations when they are receptive, and female guppies are normally receptive when they are virgin, recently mated (up to 5 days), or in a postpartum phase (Guevara-Fiore et al. 2009). We used a total of 28 virgin females, from an unselected base population, and these females were changed twice per day to maintain their receptive status. The procedure was repeated for 14 consecutive days because guppies from high-predation populations have been shown to learn the way through a simple maze within 2 weeks (Burns and Rodd 2008), and the origin of the here used animals is a high-predation site (Kotrschal et al. 2013a). Failure to reach the female within the 8-min period was only observed during the first 3 days and for a limited number of males (day 1: 6 large-brained and 6 small-brained individuals, day 2: 6 large-brained and 6 small-brained individuals, and day 3: 2 large-brained and 3 small-brained individuals). This almost identical number of large- and small-brained males not finishing the maze within the 8-min period means that exclusion of those males is unlikely to have created a systematic bias in our results.

### Validation of brain size difference

F3 males were used for the current experiment. Therefore, to determine that they showed the relative brain size differences previously demonstrated in F2 fish for these selection lines, we conserved them in 4% paraformaldehyde (buffered in phosphate-buffered saline) after the experiment and measured their standard length and brain weights analogously to Kotrschal et al. (2013a).

### Statistical analyses

We used a general linear mixed model (GLMM) to test for a potential impact of brain size on the time the fish took to find the female over the course of the experiment. To investigate this in the large- and the small-brained lines, we included “search time” (log10 transformed to meet normality), that is, the time from leaving the start compartment until reaching the end compartment of the maze as dependent variable, brain size selection line as fixed effect, replicate and individual as random effects, and day of experiment and (day of experiment)^2^ (to test for nonlinear associations) as covariates. To investigate potential differences in the learning curve between selection lines, we analyzed the interaction between brain size selection line and day of experiment. To test whether large- and small-brained fish differed in the time to find the female after a learning period, we used an analogous GLMM with the search time of the last 3 days as dependent variable. Because personality may influence how fast individuals explore a novel environment like the maze in this setup (Bolhuis et al. 2004), we compared the latency until the fish left the start compartment on day 1 as an indicator of personality. To analyze this aspect, we used an analogous GLMM to the one described above. In order to estimate the motivation to find a mate, we analyzed the latency after day 3 (e.g., after all fish had learnt to navigate the maze without guidance). To determine the relative brain size of the males used in this experiment, we used a GLMM with brain weight as dependent variable, body size as covariate, brain size selection line as fixed effect, and replicate as random effect. Nonsignificant interactions (*P* > 0.3 in all cases) were excluded in all models. Note that the size of the data set prevented residual distributions from being perfectly normal despite transformation; they were always biased toward more central estimates. All analyses were done in SPSS 19.0.

### Ethical statement

Our research followed the ABS/ASAB Guidelines for the Use of Animals in Research and was approved by the Uppsala Ethical board (Ethical permit C50/12).

## RESULTS

**Figure 2:**
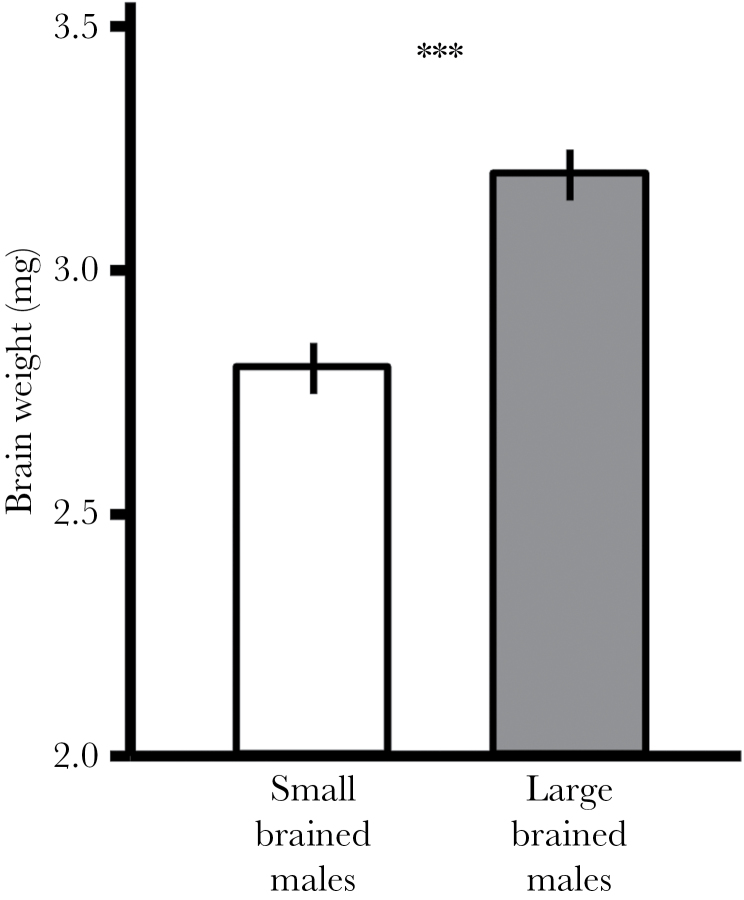
F3 male guppies (*Poecilia reticulata*) selected for large relative brain size showed 13.8% heavier brains than males selected for small relative brain size. Shown are the estimated marginal means (mg ± SE) of a GLMM calculating brain weights of the fish used in this experiment corrected for body size and replicate. ***P < 0.001.

The subsample of animals chosen for this experiment from the large- and small-brained selection lines differed by 13.8% in relative brain size (GLMM: brain size selection line: *F*_1,28_ = 26.9, *P* < 0.001; body size: *F*_1,28_ = 51.1, *P* < 0.001, Figure 2).

**Figure 3:**
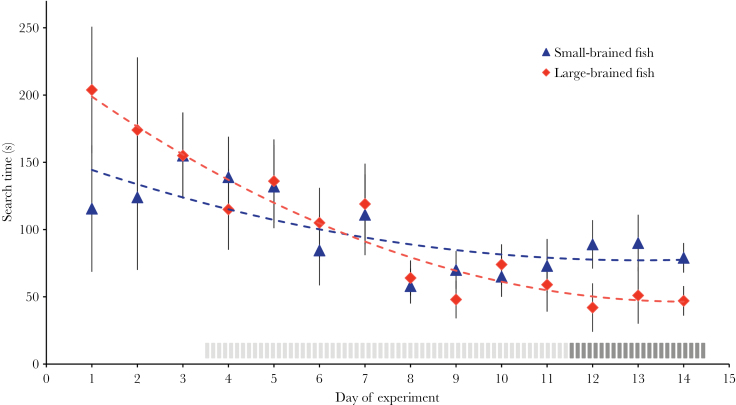
Maze learning in male guppies (*Poecilia reticulata*) selected for large and small brain size. Large-brained males (red diamonds) are faster to learn the way through a maze than small-brained males (blue triangles). The dashed gray area indicates the days when all fish finished the maze without guidance; the dark gray area marks the days when large-brain animals were faster to find the female than small-brain males. Shown are the estimated marginal means of 14-day-specific GLMMs with search time as dependent variable, controlled for replicate (s ± SE). Note that depicted data are untransformed, whereas data used in the analyses were log10 transformed (see main text).

During the 2 weeks of the experiment, both large- and small-brained fish significantly decreased the time through the maze to find the female (“search time,” GLMM_day1–14_: day of experiment: *F*_1,34_ = 16.5, *P* < 0.001; (day of experiment)^2^: *F*_1,428_ = 5.9, *P* = 0.016, Figure 3). The decrease in search time was significantly steeper in the large-brained fish (GLMM_day1–14_: day of experiment × brain size selection line: *F*_1,34_ = 5.9, *P* = 0.016, Figure 3). There was a nonsignificant tendency for the intercept of the learning curve of large-brained fish to be higher (GLMM_day1–14_: *F*_1,259_ = 3.1, *P* = 0.081, Figure 3). At the end of the experiment, when the animals had learnt their way through the maze, large-brained males were on average nearly twice as fast at finding the female than the small-brained males (mean search time: 86±14 vs. 47±14 s; GLMM_day12–14_: brain size selection line: *F*_1,99_ = 9.4, *P* = 0.003). We note that the interaction between day of experiment and brain size in the overall model was not merely driven by a potential difference in search time on day 1 because a model excluding day 1 showed qualitatively similar results (GLMM_day2–14_: brain size selection line: *F*_1,34_ = 1.4, *P* = 0.235; day of experiment: *F*_1,34_ = 16.5, *P* < 0.001; (day of experiment)^2^: *F*_1,34_ = 7.7, *P* = 0.006; day of experiment × brain size selection line: *F*_1,34_ = 3.6, *P* = 0.064). Moreover, and importantly, search time on day 1 did not differ between groups (GLMM_day1_: *F*_1,22_ = 2.0, *P* = 0.168). The faster decrease in search time in large-brained compared with small-brained animals should, therefore, be driven by differences in learning ability and not by any preexisting differences, such as a different response to a novel situation. In fact, in this context, males of different brain sizes did not differ in personality because on the first day of the experiment, they did not differ in their latency to leave the start compartment (large brains = 129±30 s, small brains = 141±28 s; GLMM_day1_: brain size selection line: *F*_1,21_ = 0.1, *P* = 0.800).

Once all fish had learnt to navigate the maze without guidance, they gradually decreased latency, but large- and small-brained fish did not differ in overall latency (GLMM_day4–14_: brain size selection line: *F*_1,33_ = 1.47, *P* = 0.234; day of experiment: *F*_1,347_ = 4.84, *P* = 0.028), suggesting no motivational differences. Furthermore, because the mean search time during the last 3 days was more than 7 times (86 and 47 s; see above) than the predicted mean average time based on previous analyses on these lines (7.7 s; see Methods), the difference in maze performance is unlikely to be caused by differences in swimming ability between the large- and small-brained lines.

## DISCUSSION

Our results show that selection on brain size results in a correlated response in spatial maze learning ability in the male guppy. Our assay demonstrated that, although males from both selection regimes exhibited the capacity to learn to find their way through the maze, large-brained males reduced the overall search time through the maze more rapidly than small-brained males. Consequently, although the groups did not differ significantly in search time at the beginning of the experiment, the large-brained individuals ended up being significantly faster in finding the females during the final 3 days.

Here, we suggest that the experimental change in brain size—via artificial selection—changed cognitive ability in the large-brained compared to the small-brained males, and so led to the observed difference in maze performance. But before accepting this explanation, it is important to consider also potential alternative explanations for our results. One potential alternative explanation for the faster learning of the route through the maze in large-brained males may be a difference in personality between large- and small-brained fish (Kotrschal et al. 2014). This is so because a more proactive personality type should habituate to a novel situation faster (the maze) and may be expected to reach the female faster than a more reactive personality type. However, on the first day of the experiment, we found neither a difference in latency nor in search time (in fact large-brained males seemed to have taken longer, a result that supports a previous finding—Burns and Rodd 2008—albeit not significantly so in our study). Although we cannot completely rule out the influence of different personality in the large-brained versus small-brained males, this speaks against a prominent role of personality affecting the results in this experiment.

Another potential alternative explanation for the difference in performance between the large- and small-brained males may be differences in motivation between the large- and the small-brained lines (Wise 2004). We suggest this is unlikely because once the focal males had learned that there was a female waiting at the end of the maze (when all fish finished the maze without guidance from day 4 and onward), the latency time to leave the start compartment did not differ between the brain size selection lines. Moreover, we have no a priori reason to believe that the large-brain males should be more motivated to find a female. In fact, large-brained individuals have been found to be less fecund in these selection lines (Kotrschal et al. 2013a). Also, large-brained males tend to be more colorful and thus potentially more attractive (Kotrschal A, Corral-Lopez A, Zajitschek S, Immler S, Maklakov AA, Kolm N, unpublished data). Therefore, if a bias in motivation existed, we would expect it to go in the opposite direction, that is, that small-brained males should compensate for their lack of attractiveness by increasing their mate search effort and so find the females faster (Kokko and Wong 2007). However, we did not observe this pattern in our study.

Finally, differences in swimming performance could have led to the faster search time during the last 3 days of the experiment in large-brained compared to small-brained individuals. However, in a recent series of swimming performance trials on large- and small-brained guppies, we found no differences in maximum swimming speed (burst swimming), endurance speed (as measured in a swim tunnel), or voluntary swimming speed (when exploring a novel arena) (Kotrschal et al. 2014). Hence, differences in swimming performance between large- and small-brained individuals are unlikely to explain the results in the maze.

Although we need to be careful in our interpretations concerning the exact mechanism behind the apparent link between brain size and cognition in this experiment, our results lend support to the hypothesis that in male guppies, relative brain size is positively associated with at least one potential aspect of cognitive ability—the ability to learn the route to a potential mate in a maze. As explained above, if these patterns are general, larger brains may yield fitness advantages in the wild because access to females is considered the major limiting factor of a male guppy’s mating success (Houde 1997). Spatial cognitive ability may even be directly related to mating success, as suggested in a recent study where female guppies were shown to prefer males that were faster at learning the way through a maze (Shohet and Watt 2009). Evidence is accumulating in support of mate choice for cognitive traits playing an important role for brain evolution both in humans (Miller 2000) and in nonhuman vertebrates (see review by Boogert et al. 2011).

Recently, the potential positive association between brain size and cognitive abilities has been challenged in light of the high cognitive capacity of some small-brained invertebrates such as bees or ants (Chittka and Niven 2009). Also, rather than the size of the brain per se, numerous fine-scale structural differences in the brain have been identified as the main features linking brain morphology and cognition (Toga and Thompson 2005). Yet, together with the previous finding of better numerical learning ability in large-brained female guppies (Kotrschal et al. 2013a), our results further corroborate that, at least on the within-population level and in this context, larger brains really can be better and that variation in a relatively crude measure of brain morphology, relative brain size, is directly associated with cognitive advantages. It will be most interesting to see if future experimental studies on the association between brain size and cognitive ability in other taxa will support our results on the guppy brain size selection lines.

The substantial costs of developing and maintaining a larger brain have made it difficult to explain how large brains evolve in wild populations (Striedter 2005). Our results support that selection for increased cognitive ability might drive the evolution of larger brains in wild populations. We, therefore, propose that the remarkable variation in vertebrate brain size that is evident at all taxonomic levels is generated through different optima (likely driven by differences in ecology), which are dictated by the balance between energetic costs and cognitive benefits of larger brains. Because cognition is the process by which an individual animal receives, learns, and remembers information in form of explicit and implicit representations (Braithwaite 2006; Shettleworth 2010), cognition consists of 3 interacting aspects: perception, learning, and memory. Whether a large brain confers benefits in 1, 2, or all 3 of those aspects and so provides the cognitive advantage demonstrated in the previous (Kotrschal et al. 2013a, 2013b) and this study will need to be clarified in future experiments.

## FUNDING

A.K. was funded by the Carl Tryggers Stiftelse (to N.K.) and the Austrian Science Fund (J 3304-B24 to A.K.). N.K. was funded by the Swedish Research Council.
